# Crossbreeding of Yeasts Domesticated for Fermentation: Infertility Challenges

**DOI:** 10.3390/ijms21217985

**Published:** 2020-10-27

**Authors:** Nobuo Fukuda

**Affiliations:** Biomedical Research Institute, National Institute of Advanced Industrial Science and Technology (AIST), 1-8-31 Midorigaoka, Ikeda, Osaka 563-8577, Japan; nob-fukuda@aist.go.jp

**Keywords:** crossbreeding, spore viability, sporulation efficiency, yeasts domesticated for fermentation

## Abstract

Sexual reproduction is almost a universal feature of eukaryotic organisms, which allows the reproduction of new organisms by combining the genetic information from two individuals of different sexes. Based on the mechanism of sexual reproduction, crossbreeding provides an attractive opportunity to improve the traits of animals, plants, and fungi. The budding yeast *Saccharomyces cerevisiae* has been widely utilized in fermentative production since ancient times. Currently it is still used for many essential biotechnological processes including the production of beer, wine, and biofuels. It is surprising that many yeast strains used in the industry exhibit low rates of sporulation resulting in limited crossbreeding efficiency. Here, I provide an overview of the recent findings about infertility challenges of yeasts domesticated for fermentation along with the progress in crossbreeding technologies. The aim of this review is to create an opportunity for future crossbreeding of yeasts used for fermentation.

## 1. Introduction

Sexual reproduction is ubiquitous in eukaryotes and expands the genetic diversity by producing progeny that resemble their parents but are NOT identical to them [1]. The benefits of sexual reproduction include the purging of deleterious mutations from the genome. Due to genetic recombination and diversification, sexual reproduction can provide a recombinant progeny, well-adapted to a changing environment [2], although there is a twofold cost of sex in relation to asexual reproduction [3,4,5,6]. It is rational that numerous species in nature maintain sexual reproduction for survival.

It is known that many species can reproduce both asexually and sexually. Budding yeasts also exhibit asexual and sexual reproductive cycles, whereas the most common mode of vegetative growth is asexual reproduction in both haploid and diploid cells. In haploid cells, there are two cell types determined by the co-dominant **a** and α alleles, at the *MA*ting *T*ype (*MAT*) locus [7,8]. While haploid cells can be annihilated under high-stress conditions such as nutrient starvation, diploid cells can endure harsh environments by undergoing sporulation during sexual reproduction (meiosis). Through a series of meiotic divisions, the four resulting haploid progenies are packaged into individual spores [9]. Opposite mating type haploid cells mate with each other to reform diploid cells, following spore germination. Similar to that of other fungi, many yeast species preserve sexual reproduction although it is not obligatory [10].

Fermentation of carbohydrate sources into ethanol using yeast species is one of the oldest technologies. The earliest evidence about the existence of a fermented beverage dates to the Neolithic period [11]. As is widely known, various yeast species are still used for many essential biotechnological processes including the production of beer, wine, and biofuels. *Saccharomyces cerevisiae* is one of the most extensively distinguished yeast species and the preferred host for many bioprocesses in large scale fermentations due to its fast growth, well-developed genetics, and fermentation robustness [12]. To increase the productivity and quality, strenuous efforts have been made to generate custom-engineered strains for each fermentation process.

Crossbreeding provides an attractive approach that allows the use of sexual reproduction to generate novel yeast strains exhibiting combined preferred characteristics [13,14,15]. Similar to that in plants and animals in the agriculture and livestock industry, crossbreeding has been traditionally used for yeast trait modifications in the brewing industry. Unfortunately, however, many industrial strains of *S. cerevisiae* are known to have infertility challenges that manifest as poor sporulation efficiency and spore viability [8,16]. In a previous study that tested the capacity of all 318 industrial yeast strains, it was found that >40% of the strains did not form spores at all [17]. Despite these infertility challenges, they are preserved as useful organisms with excellent fermentation characteristics in human societies. Here, I review the recent findings about infertility challenges of yeasts domesticated for fermentation, for the future consideration of crossbreeding in the brewing industry. The *S. cerevisiae* genes described in this review are summarised in Table 1.

## 2. Sex Determination and Behaviour in Budding Yeasts

Mating of the opposite mating type haploid cells (*MAT***a** and *MAT*α) results in the formation of a new diploid strain (*MAT***a**/α). Phenotypic differences arise from the different gene expression patterns regulated by the *MAT* loci. The differences in gene expression are summarized in Table 2. Genes involved in mating are divided into three categories that include **a**-type-specific genes (***a****sg*), α-type-specific genes (*αsg*), and haploid-specific genes (*hsg*) [32]. *MAT***a** haploids express the genes ***a****1* whereas *MAT*α haploids express the genes *α1* and *α2*. The synthesized protein α1 is the activator of αsg, and α2 functions as a repressor of asg. While the synthesized **a**1 protein does not influence the expression of mating-related genes, the formation of **a**1-α2 complex suppresses the expression of α1 and *hsg* in *MAT***a**/α diploid cells.

**Figure 1:**
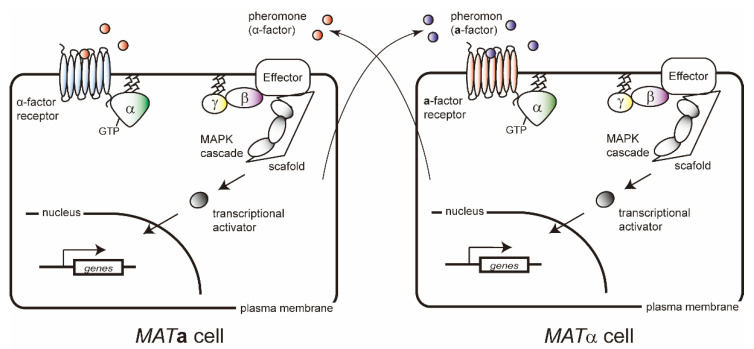
Signal transduction pathway of budding yeasts in response to mating pheromones. Mating pheromones, **a**-factor and α-factor, are secreted from *MAT***a** and *MAT*α cells, respectively. Binding of the mating pheromones to a seven-transmembrane, G-protein-coupled receptor (GPCR) on the cell-surface, leads to activation of heterotrimeric G-proteins composed of Gα, Gβ, and Gγ. The activated G-proteins subsequently dissociate into Gα and a Gβγ dimer, and then Gβγ dimer induces activation of the mitogen-activated protein kinase (MAPK) cascade, resulting in mating-responsive gene expression.

In *S. cerevisiae*, the process of mating is regulated by a single canonical G-protein signaling pathway [33]. Most of components in this pathway are shared between *MAT***a** and *MAT*α cells (Figure 1). *MAT***a** cells express the G-protein coupled receptor (GPCR) Ste2, which detects the α-factor mating pheromone expressed from the *MFα1* gene in *MAT*α cells. Reciprocally, *MAT*α cells express the receptor Ste3, which binds with the **a**-factor pheromone expressed from the *MFA1* gene in *MAT***a** cells. Pheromone stimulation leads to the activation of heterotrimeric G-proteins comprising Gpa1 (Gα), Ste4 (Gβ) and Ste18 (Gγ) through the GPCR. The activated G-protein subsequently dissociates into Gα and Gβγ complex subunits, and the Gβγ complex induces activation of the mitogen-activated protein kinase (MAPK) cascade [19] followed by transcriptional activation for cell fusion (Figure 1). While the G-protein subunit genes *GPA1*, *STE4*, and *STE18* are *hsg*, the pheromone and receptor genes are ***a****sg* or *αsg* [18,34]. Expression of these genes is regulated by the *MAT* gene, resulting in mating behaviors between the *MAT***a** and *MAT*α cells. In the generated *MAT***a**/α diploid cells, pheromone response signal transduction pathway does not function due to the lack of components.

**Figure 2:**
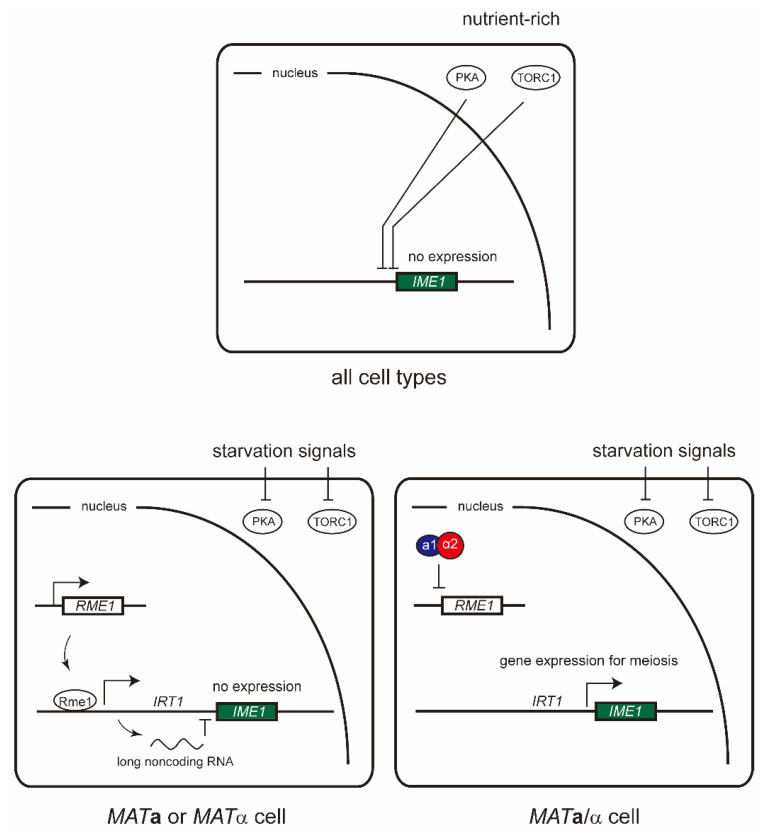
Meiosis is induced by the expression of the *I*nducer of *ME*iosis 1 (*IME1*) gene which is regulated by both mating type and nutrient starvation. In nutrient-rich environments, *IME1* is suppressed by the activities of target of rapamycin complex I (TORC1) and protein kinase A (PKA). While *IME1* expression is activated by starvation signaling, it is also repressed by the gene products of the *R*egulator of *ME*iosis 1 (*RME1*) and *I*ME1 *R*egulatory *T*ranscripts (*IRT1*). In both haploid cells (*MAT***a** and *MAT*α), the absence of the **a**1-α2 complex permits expression of the *RME1* gene. Then Rme1 transcribes a long noncoding RNA, the product of *IRT1*, which in turn represses *IME1*. The **a**1-α2 complex directly represses the expression of the *RME1* gene, and the gene product of *IME1* activates the meiosis pathway in *MAT***a**/α diploid cells in response to starvation.

*MAT***a**/α diploid cells can undergo meiosis under starvation conditions, while *MAT***a** and *MAT*α haploid cells cannot undergo meiosis when exposed to such an environment. Meiosis is regulated by the expression of the *I*nducer of *ME*iosis 1 (*IME1*) gene, commonly known as the master initiator of meiosis [20]. In nutrient-rich environments, *IME1* is suppressed by two major nutrient sensing signaling pathways [21,35] (Figure 2). Target of rapamycin complex I (TORC1) and protein kinase A (PKA) activities are suppressed in the absence of nitrogen and glucose. *IME1* expression is activated by starvation signaling and repressed by the gene products of the *R*egulator of *ME*iosis 1 (*RME1*) and *I*ME1 *R*egulatory *T*ranscripts (*IRT1*) [21]. The difference in gene expression (Table 1) is a result of differential gene regulation by the **a**1-α2 complex. In both *MAT***a** and *MAT*α haploid cells, the absence of the **a**1-α2 complex induces expression of the *RME1* gene, a member of *hsg*. Rme1 is a zinc finger protein that activates expression of a long noncoding RNA, the product of *IRT1*, which in turn represses *IME1*. Meiosis is not induced by starvation signaling in haploid cells, as the expression of the *IME1* gene is repressed. In contrast, the **a**1-α2 complex directly represses the expression of the *RME1* gene, and the gene product of *IME1* activates the meiosis pathway in *MAT***a**/α diploid cells in response to starvation. It should be noted that the **a**1-α2 complex is the most important factor distinguishing *MAT***a** and *MAT*α haploids from *MAT***a**/α diploids.

## 3. Mating Type Switching and Autodiploidization

Whereas cell type determination in many multicellular organisms is an irreversible process, the mating types of haploids (**a** and α) of the *Saccharomycetaceae* yeast family can interconvert in a reversible manner by a programmed DNA-rearrangement process called mating type switching [18,22]. Following spore germination, many haploid cells mate with the nearby cells of the opposite mating type. In contrast, to mate and become diploid cells, haploid cells that cannot contact the mating partners convert sister cells from one mating type to the other. The endonuclease Ho is responsible for the mating type switching in *S. cerevisiae*. Following DNA cleavage at the *MAT* locus, replacement of **a** or α DNA sequences is induced by the opposite sequences derived from one of the two silent donor loci, *HMR***a** and *HML*α [36]. It is known that loss-of-function mutations in the *HO* gene (*ho* allele) prevent mating type switching of the haploid cells [37]. Yeast strains containing the *ho* allele are categorized into heterothallic yeasts, whereas homothallic yeasts maintain the *HO* gene inducing mating type switching in the haploid cells.

**Figure 3:**
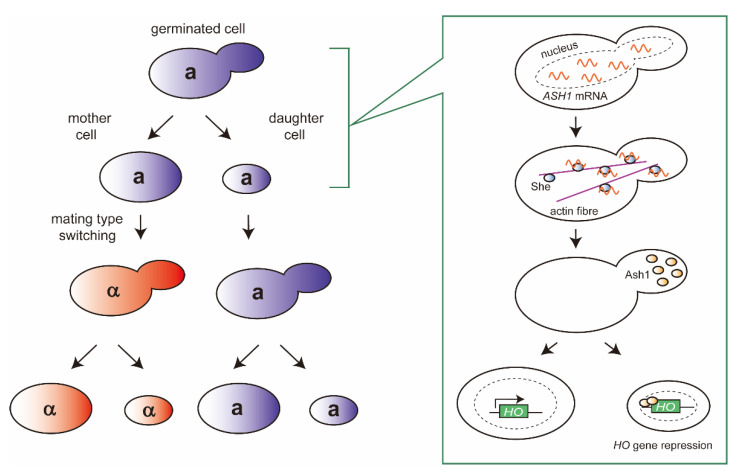
Asymmetric expression of the *HO* gene between mother and daughter cells. Mating type switching occurs exclusively in the mother cells, and never in the daughter cells. Mother/daughter asymmetric *HO* gene expression is induced by the product of the *HO*-specific repressor gene, *A*symmetric *S*ynthesis of *H*O (*ASH1*). *ASH1* mRNA is delivered to the daughter cells by the products of the *S*wi5p-dependent *H*O *E*xpression (*SHE*) genes. She proteins bind to *ASH1* mRNA in the nucleus, mediate export to the cytoplasm, and transport them along the actin fiber to the bud tip. In the daughter cells, Ash1 binds its consensus sequences within the upstream repression sequence 1 (URS1) of the *HO* gene in the following G1 phase. Meanwhile the endonuclease Ho is exclusively synthesized in the mother cells, resulting in mating type switching.

The *HO* gene is a member of *hsg* and is repressed by the **a**1-α2 complex in the diploid cells. In addition, mating type switching occurs exclusively in the mother cells, and never in the daughter cells (Figure 3), due to the difference in *HO* gene expression. Mother/daughter asymmetric *HO* gene expression is induced by the product of the *HO*-specific repressor gene, known as the *A*symmetric *S*ynthesis of *H*O (*ASH1*) [23]. Delivery of the *ASH1* mRNA to the daughter cells is achieved by the products of the *S*wi5p-dependent *H*O *E*xpression (*SHE*) genes. She2 binds to *ASH1* mRNA in the nucleus and mediates export to the cytoplasm, after which She3 associates with the ribonucleoprotein particle (RNP). She proteins act as adapters for the type V myosin Myo4 (also called She1), transporting the RNP complex along the actin fiber to the bud tip [24]. *ASH1* mRNA is finally translated in the daughter cells, and Ash1 binds its consensus sequences within the upstream repression sequence 1 (URS1) of the *HO* gene in the following G1 phase. Meanwhile the endonuclease Ho is exclusively synthesized in the mother cells (only during late G1), resulting in mating type switching. Using this programmed DNA-rearrangement, yeasts can proceed with sexual reproduction despite beginning from just a single haploid cell.

## 4. Infertility Challenges of Industrial Yeasts

### 4.1. Past and Current Situations of Yeast Sporulation

In 1996, the genome of *S. cerevisiae* was completely sequenced as the first among the eukaryotic organisms [38]. For several decades, yeast has served as a model for eukaryotic organisms, which has contributed significantly to the progress of cell biology. Compared to the non-essential genes, yeast essential genes exhibited more homologs in the other organisms [39,40]. Importantly, tetrad spore analysis revealed 1105 essential genes of *S. cerevisiae*, based on whether it is possible to acquire haploid cells with the target genes [40,41]. In fact, yeast strains without sporulation deficiency have been utilized for these analyses.

In industrial use, sporulation invites great attention as a means of crossbreeding. Unfortunately, many industrial *S. cerevisiae* strains are known to exhibit poor ability for sexual reproduction. It was reported that >80% of the lager, >60% of sake, >50% of ale, and >15% of wine yeast strains did not undergo sporulation, and more than half of the spores were inviable after dissection in >80% of the lager, >30% of sake, >70% of ale, and >50% of wine yeast strains undergoing sporulation [17]. Many species of the *Saccharomyces* genus other than *S. cerevisiae* are used for industrial fermentation (including the production of lager beer, wine, and cider), some of which are interspecies hybrids [42,43,44,45] such as the lager yeast *S. pastorianus* derived from the two parental species *S. cerevisiae* and *S. eubayanus*. While these different species can mate, hybrid offspring are almost completely sterile, producing <1% viable spores [46]. The hybrid genomes can undergo extensive modifications including loss of chromosomes and various types of recombination events, resulting in aneuploidies, massive copy number variations [47]. Hybrid sterility seems to be mainly caused by the instability of the chromosomes [43].

Regardless of the defects in sexual reproduction, the generated crossbreds (mainly diploid cells) can be continued for use in ethanol fermentation by asexual proliferation. In addition, mutagenesis is also one of the conventional and effective approaches used for modifying certain traits; hence, there is a possibility that numerous industrial yeast strains have been derived from a few strains with defects in sexual reproduction. In modern human societies, industrial yeasts do not need to withstand harsh environmental changes if they maintain excellent characteristics during fermentation. In fact, yeast strains with higher fermentation rates have been historically selected to shorten the fermentation periods and spontaneously inhibit the growth of undesirable microorganisms. Recently, it was found that gene mutations in nutrient-regulated genes are related to high fermentation rates [48].

### 4.2. Loss-of-Function Mutations in Nutrient-Regulated Genes

Sake yeast strains produce significantly more ethanol during fermentation than any other type of *S. cerevisiae* strains. Gene expression profiling has revealed that the stress-responsive transcription factors Msn2 and Msn4 (Msn2/4) are inactivated in sake yeast strains during fermentation [25,26]. It is known that sake yeast strains are more sensitive to ethanol stress and heat shock than the laboratory strains. Dysfunction of Msn2/4 increases the fermentation rates through the modification of glucose metabolism, including reduced synthesis of storage and structural carbohydrates [48]. Although a specific loss-of-function mutation was found in the *MSN4* gene (*msn4^C1540T^*) of sake yeast strains, this mutation does not appear to be solely responsible for the inactivation of Msn2/4 [43]. In addition, deletion of the *MSN4* gene in the *S. cerevisiae* laboratory strains leads to only a modest increase in their fermentation rates [26]. The inactivation of Msn4 is inadequate to achieve the high fermentation rate observed in sake yeasts, suggesting the existence of an alternative factor that inhibits Msn2/4 activities and contributes to the brewing properties.

**Figure 4:**
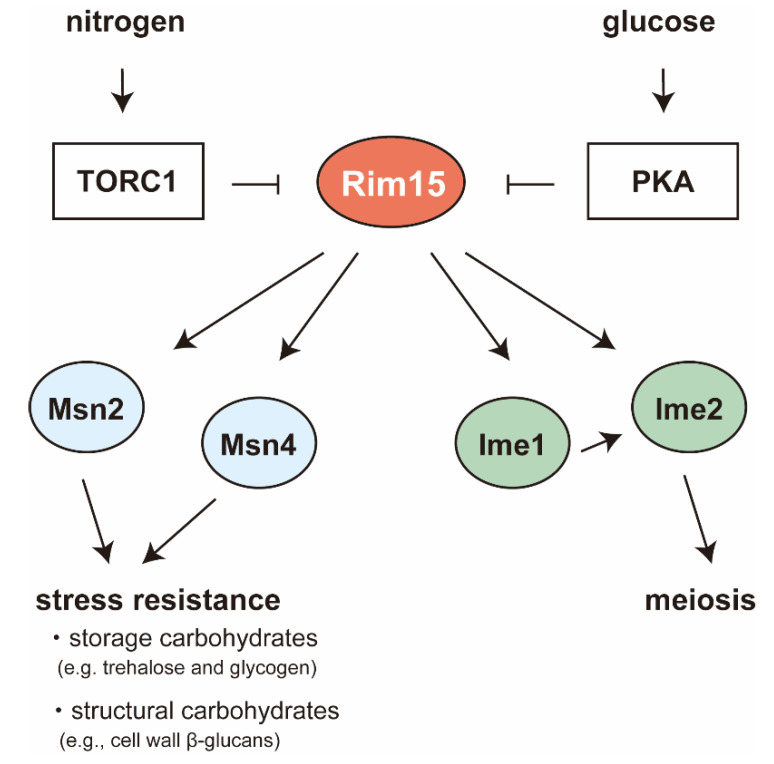
Regulatory cascades around the PAS-kinase Rim15. Rim15-responsive transcripts are involved in stress resistance, carbohydrate metabolism, and respiration. Meanwhile, in response to nutrient starvation, *RIM15* contributes to gene expression of the *IME1* gene, and helps *IME1* activate the downstream sporulation genes such as *IME2*.

In *S. cerevisiae*, nutrient starvation causes inactivation of the nutrient-sensory kinases such as PKA and TORC1, resulting in activation of the PAS kinase Rim15 (Figure 4). *RIM15* was identified as one of functionally related genes (*R*egulator of *IM*E2) that stimulate early meiotic gene expression in yeast [27]. In response to nutrient starvation, *RIM15* contributes to gene expression of the *IME1* gene [49] and helps *IME1* activate the downstream sporulation genes such as *IME2* [28,29]. Meanwhile, Rim15-responsive transcripts are involved in stress resistance (essentially heat shock and oxidative stress resistance), carbohydrate metabolism, and respiration [50]. Most of these genes are regulated by the transcription factors, Gis1 binding to the post-diauxic shift (PDS) element, and Msn2/4 recognizing the stress response element (STRE) [30]. Rim15 has been known to be involved in the activation of Msn2/4 (Figure 4) by phosphorylating both N-terminal (1–400) and C-terminal domain (401–704) of Msn2 [51].

In sake yeast strains, the insertion of a single adenine nucleotide was found immediately after position 5067, compared to that in *S. cerevisiae* laboratory strains. The frameshift mutation at nucleotide position 5068 (*rim15^5055insA^*) generates a premature stop codon that shortens the *RIM15* gene product by 75 amino acids in the C-terminal region [48], which severely impairs the function of Rim15. Since sake yeast strains contain both copies of the *rim15^5055insA^* mutations, they exhibit a high fermentation rate, stress sensitivity, and severe defects in meiosis. It might be inevitable that infertility challenges are broadly seen in industrial yeasts selected by using fermentation characteristics as the indicator. By introduction of the wild type *RIM15* in the sake yeast strains, the sporulation efficiency significantly increased; however, they produced few viable spores [16].

### 4.3. Chromosome Recombination Defect in Meiosis

**Figure 5:**
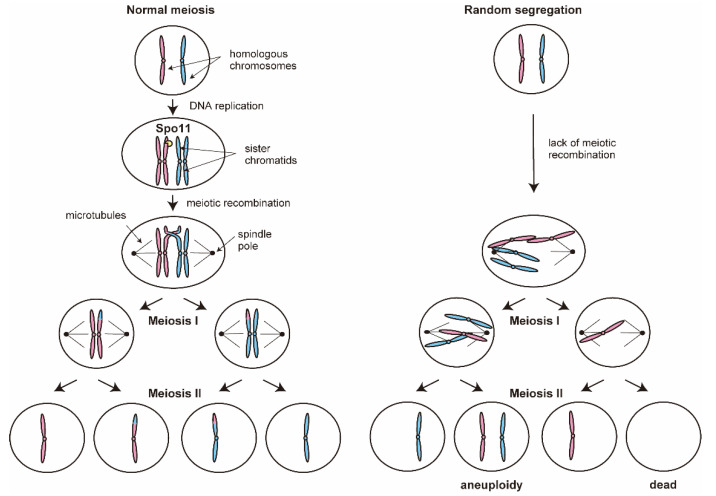
The role of chromosomal recombination in meiosis. Homologous parental chromosomes pair and separate at meiosis I, while sister chromatids segregate during meiosis II. Spo11 is a meiosis-specific protein that initiates meiotic recombination, and the deletion of *SPO11* gene completely prevents linkage of the homologous chromosomes, resulting in random segregation of homologous parental chromosomes at meiosis I.

In normal meiosis, the diploid genome is reduced to produce haploid gametes through two rounds of nuclear division that follow a single round of DNA replication (Figure 5). Homologous parental chromosomes pair and separate at the first cell division (meiosis I), while sister chromatids segregate during the second division (meiosis II). One of the main reasons for poor spore viability in industrial yeast strains is a chromosome recombination defect in meiosis. Since meiotic recombination is an essential step for linkage of the homologous parental chromosomes, its absence leads to uneven inheritance of the chromosomes (nondisjunction) leading to aneuploidy and inviable gametes [16]. Spo11 is a meiosis-specific protein that initiates meiotic recombination by catalyzing the transesterification reaction in DNA double-strand breaks. The deletion of the *SPO11* gene completely abolishes meiotic recombination, and prevents linkage of the homologous chromosomes, resulting in random segregation of homologous parental chromosomes at meiosis I [31,52].

Compared to that in *S. cerevisiae* laboratory strains, there are several mutations in the *SPO11* gene of sake yeast strains. The single nucleotide substitution of C73T (*spo11^C73T^*) that results in the R25W missense mutation is found in sake yeast strains with significantly poor spore viability. Introduction of *spo11^C73T^* to the laboratory strain causes sporulation deficiency and spore non-viability, whereas the transformants with other mutations except for C73T show a high sporulation efficiency and produce many viable colonies [16]. As described above, many sake yeast strains also contain loss-of-function mutations in the nutrient-regulated genes. Therefore, in these strains, introduction of the intact *SPO11* cannot completely resolve the infertility challenges. In fact, introduction of both intact *RIM15* and *SPO11* leads to high sporulation efficiency and spore viability [16].

### 4.4. Difficulty and Contradiction in Conventional Crossbreeding

Whole genome and transcriptome analyses have revealed genetic differences between strains of the same species. It was reported that mutations in >200 genes affect yeast sporulation [53,54]. They have been divided into the following five categories according to gene functions [55,56,57]: (i) mitochondria/metabolism, (ii) vacuolar and autophagy, (iii) meiosis/early sporulation, (iv) spore formation, and (v) undefined role in sporulation. Mutants in the first two categories arrest the process prior to the meiotic divisions. Sporulation is triggered by nitrogen starvation and requires the presence of a nonfermentable carbon source [58]. The nonfermentable carbon source is metabolized to produce energy via the Krebs cycle and to provide precursors of macromolecules such as nucleic acids, lipids, and polysaccharides required for spore formation. Therefore, respiratory-incompetence blocks entry into sporulation [59,60]. Similarly, because new protein synthesis during sporulation requires the recycling of preexisting proteins, many genes involved in delivery of proteins to the vacuole or in autophagy contributes to entry into the meiotic divisions.

In natural selection, these mutations might be purged from the yeast genome. Unfortunately, however, it is not easy to remove the deleterious mutations for sporulation from industrial yeast strains. To begin with, screening methods corresponding to each specific phenotype of interest are usually required for the introduction of a specific change at the target genes. In the case of a phenotype involved in meiosis, observation of individual sporulation consumes immense time to evaluate the clones that have been generated and isolated from the cell population. In addition, some meiosis-related genes such as *RIM15* interfere with the fermentation characteristics that need to be improved mainly in the breeding of industrial yeasts. Removal of mutations in these genes inevitably causes a decline in productivity of the fermentation processes without any improvement in the product quality.

Spore viability in interspecific hybrids further declines due to nucleotide divergence [61,62]. The *Saccharomyces* genus is composed of eight species (*S. arboricola*, *S. cerevisiae*, *S. eubayanus*, *S. jurei*, *S. kudriavzevii*, *S. mikatae*, *S. paradoxus* and *S. uvarum*) [63,64,65,66], and some studies demonstrated that interspecific hybridization can also occur in nature [67,68]. While industrial *Saccharomyces* hybrids inherit good fermentation performance such as growth ability at lower temperatures [69,70,71,72,73], hybridization gives rise to instability of the chromosomes, which in turn results in spore unviability [74,75]. It has been also reported that sporulation efficiency is significantly anticorrelated with the fraction of the genome associated with large (>20 kb) amplifications and deletions [76]. Aneuploidies and massive copy number variations are also seen in inter-strain hybrids of *S. cerevisiae*. A ploidy study revealed that sake yeast strains (belonging to *S. cerevisiae*) are mostly diploids, but some are aneuploid [77]. Regarding the effect of chromosomal aneuploidy on the brewing characteristics, it has been reported that trisomy of chromosomes XI and XIV leads to the pyruvate underproduction [78]. Unfortunately, although a variety of yeast strains have been generated using it, the technique of conventional crossbreeding has reached its limit in trait modification of yeast strains with favorable brewing characteristics. To meet the needs of future yeast strain development in industrial use, alternative techniques to bypass sporulation are essentially required to acquire mating-competent yeast cells.

## 5. Alternative Techniques for Acquiring Mating-Competent Yeast Cells

### 5.1. Artificial Mating Type Conversion Using the Ho Endonuclease

**Figure 6:**
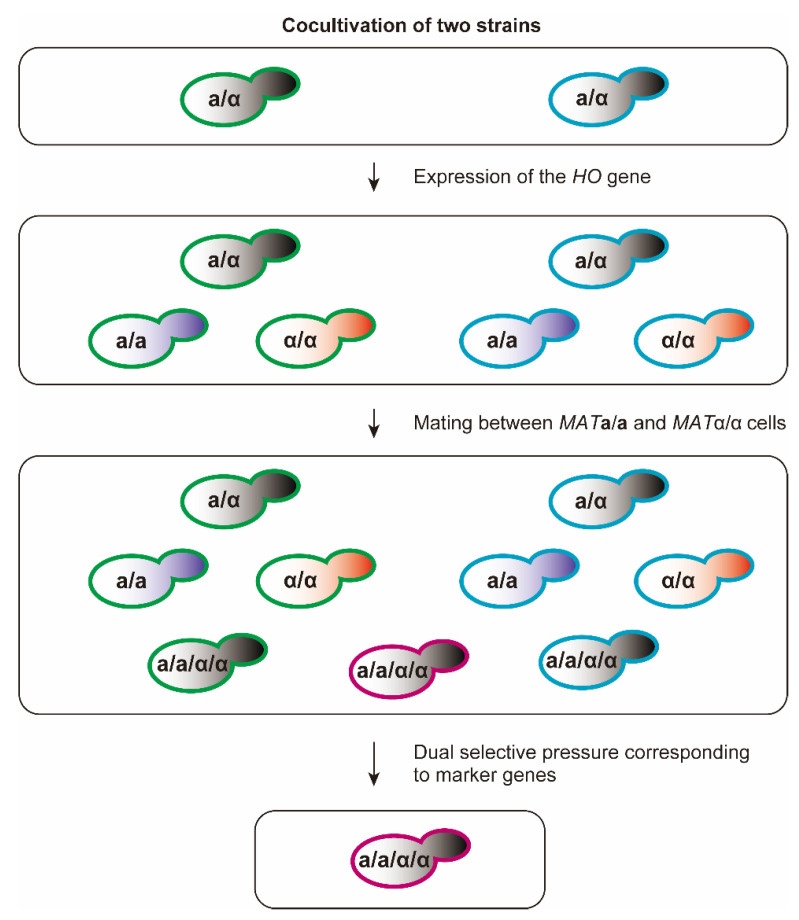
Generation and isolation of hybrid strains using artificial mating type conversion. Different marker genes are introduced into each parental strain. By the action of the Ho endonuclease, *MAT***a**/**a** and *MAT*α/α diploid cells are generated from parental *MAT***a**/α diploid cells during cocultivation, followed by formation of tetraploid cells. Green and cyan outlines indicate yeast cells possessing different maker genes, whereas purple outline indicates the inheritance of both marker genes. Crossbreeds are screened from cell population under the dual selective pressure corresponding to two marker genes inherited from parent strains.

The Ho endonuclease expressed in haploid cells of homothallic yeasts has been used for artificial mating type conversion [79]. Through forced expression of the *HO* gene, replacement of **a** or α DNA sequences at the *MAT* loci is induced by opposite mating-type sequences derived from one of two silent donor loci (*HMR***a** and *HML*α), also in *MAT***a**/α diploid cells. By the action of the Ho endonuclease, *MAT***a**/**a** and *MAT*α/α diploid cells are generated from parental *MAT***a**/α diploid cells (Figure 6), which possess the same mating ability as either *MAT***a** and *MAT*α haploids generated via sporulation. Since autopolyploidization impedes the isolation of mating-type-converted (*MAT***a**/**a** and *MAT*α/α) cells, hybrid tetraploid strains generated during cocultivation along with the artificial mating type conversion have been selected from the cell population by using marker genes introduced into each parental strain [80,81].

**Figure 7:**
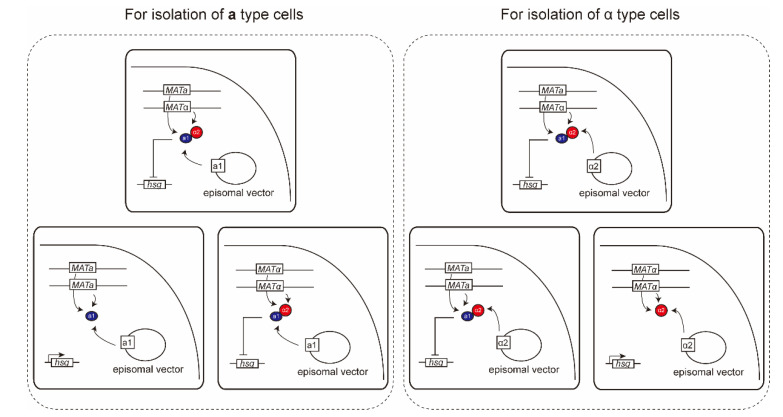
Synthetic gene expression circuit for the **a**1-α2 complex formation. To prevent autopolyploidation and isolate mating-competent cells generated in artificial mating type conversion, the **a**1 or α2 gene is introduced into parental *MAT***a**/α cells using an episomal vector.

To avoid autopolyploidization and isolate the mating-competent cells generated through artificial mating type conversion, the **a**1-α2 complex is artificially formed by introducing synthetic gene expression circuits (Figure 7) into the parental *MAT***a**/α diploid cells, using episomal vectors [36,82]. *MAT***a**/**a** or *MAT*α/α diploids generated through artificial mating type conversion are easily isolated from the cell population since the abundance ratio of target cells in the cell population reaches much beyond 20% by preventing autopolyploidization [36]. The mating type of the isolated cells can be identified by a mating assay using **a** and α tester strains [82,83,84]. Hybrid tetraploid strains are generated through cocultivation of *MAT***a**/**a** and *MAT*α/α diploids derived from different strains, and selected from the cell population by using marker genes on episomal vectors introduced into each parental strain. By the removal of episomal vectors, the introduced synthetic gene expression circuits can be rendered non-functional in yeast cells, and there is no detection of exogenous DNA throughout the entire genome of crossbreeds [12].

The *MAT* locus consists of five regions (W, X, Y, Z1, and Z2) based on the sequences shared with the two silent copies, *HML*α and *HMR***a** [85]. The difference between the **a** and α type DNA sequences exists in the Y region (Y**a** or Yα). The nucleotide sequences recognized by the Ho endonuclease reside within the Z1 regions of the *MAT* loci [86]. It is known that a single nucleotide substitution (called stuck mutation) in the Z1 region prevents mating type switching by severely reducing the Ho endonuclease cleavage. To clarify the presence or absence of stuck mutations, the *MAT* gene sequences of target strains should be confirmed in advance. Importantly, the mechanism of mating type switching is common among the *Saccharomyces* genus [22]. There is variation in the sequence of the *HO* genes between species [87,88], synthetic interspecies hybrids, *S. cerevisiae* × *S. eubayanus*, *S. cerevisiae* × *S. kudriavzevii*, and *S. cerevisiae* × *S. uvarum*, were generated as synthetic lager, Belgian, and cider strains, respectively, by introducing and expressing the *HO* gene derived from *S. cerevisiae* [80]. Furthermore, *Saccharomyces* allopolyploids of six species were similarly generated through mating type conversion of synthetic interspecies hybrids [81], which implies the applicability of these approaches to a wide range of industrial yeast strains (including natural interspecies hybrids).

### 5.2. Chromosomal Aberrations Related with Mating Type

The *MAT* genes are located on chromosome III, which is the most unstable of all 16 *S. cerevisiae* chromosomes [89]. This instability irregularly produces other sexual reproduction routes derived from chromosomal aberrations occurring during the mitotic division. There are several kinds of chromosomal aberrations such as loss of heterozygosity (LOH) and mitotic chromosome loss. In diploid cells, LOH is a naturally occurring process that generates homozygous loci through chromosomal rearrangement of the heterozygous loci [90]. LOH occurring at the *MAT* loci within *MAT***a**/α diploid cells produces either a *MAT***a**/**a** or a *MAT*α/α diploid cell, whose spontaneous frequency is less than 1 × 10^−4^ [91]. Mitotic chromosome loss is another naturally occurring event where diploid cells lose single or multiple chromosomes [92]. The loss frequency of chromosome III in yeast diploid cells has been reported at 5 × 10^−5^ [89], and yeast cells having lost one of the two copies of chromosome III acquire either **a** or α type mating ability. While the spontaneous frequencies of such events are quite low, polyploids and aneuploids can be naturally generated not by sporulation but by chromosomal aberration during mitotic division.

Rare mating based on chromosomal aberration during mitotic division can offer a way to obtain hybrid strains [93]. In a rare mating, *MAT***a**/α diploid cells can hybridize with a haploid cell of the opposite mating type following chromosomal aberration during cocultivation. The isolation of outcrossed hybrids is often achieved by using a respiratory-deficient and an auxotrophic parental strain, making rare hybrids easily selectable by their prototrophy and respiratory proficiency [94,95,96]. In addition, the isolation of both **a** and α type mating-competent cells derived from *MAT***a**/α diploid cells is achieved by using the expression of the marker gene in a mating type-specific manner [83,97]. This permits the efficient selection of **a** or α type cells from within a cell population, despite the significantly low frequency of chromosomal aberration. Meanwhile, dual-dye fluorescent staining was carried out to isolate hybrid cells without using marker genes [98,99]. By labeling each parental strain with a respective fluorescent dye prior to mating, mated cells can be enriched by fluorescence-activated cell sorting (FACS). When applied to rare mating, FACS-based selection of dual-stained cells allowed efficient enrichment of interspecies *Saccharomyces* hybrids without requiring selectable hybrid phenotypes, both for homothallic and heterothallic strains [99].

### 5.3. Conversion of the MAT Gene in Haploid Cells

Since most of the strains used in industrial use are homothallic [17,100], a haploid in the spore progeny remains only transiently, despite the success in sporulation. In wine yeast strains, conversion of homothallic strains to heterothallism was achieved by introduction of the *ho* allele [100]. Meanwhile, heterothallic yeasts containing the *ho* allele are also used in brewing. It is known that most of the modern sake yeast strains are heterothallic yeasts [101]. While facing difficulties in sporulation, many kinds of haploid strains have been generated in the process of crossbreeding. As a matter of course, pairs of the same mating type cannot mate with each other, and conversion of the mating types of either parent is a prerequisite for crossbreeding.

Similar to diploid cells, the Ho endonuclease has been used for the artificial mating type conversion of haploid cells [36,79]. However, it is difficult to convert mating types of yeast strains with stuck mutation at the *MAT* gene as described above. Recently, the *MAT* gene of haploid cells was successfully substituted with a synthetic DNA containing *MAT***a** or *MAT*α, through homologous recombination [84]. Mating type alteration of yeast cells was performed by suppressing the mating ability of parental cells in the same manner shown in Figure 7. Due to the absence of cell-cell interaction between *MAT***a** and *MAT*α cells, the mating type-altered cells independently exist in the cell population. Regardless of the stuck mutations, all haploid strains of heterothallic yeasts can be utilized for crossbreeding in any combination.

**Figure 8:**
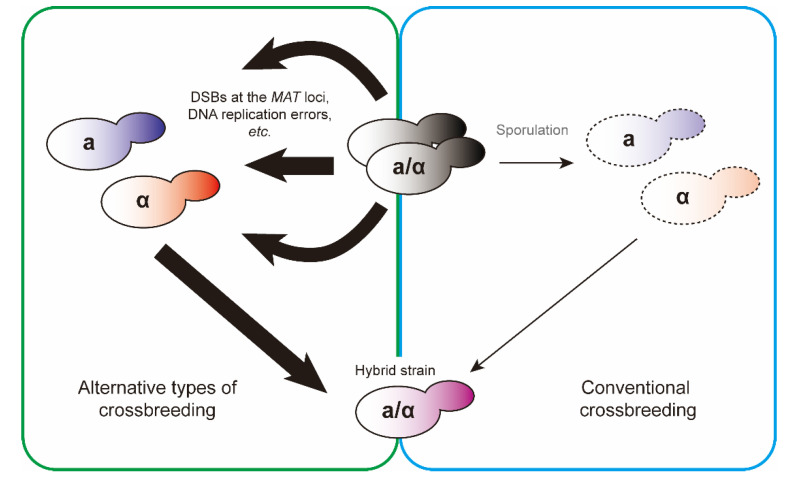
Variations of yeast crossbreeding. Since yeast strains domesticated for fermentation often have infertility challenges, it takes tremendous time and effort to generate hybrid strains in conventional crossbreeding. Meanwhile, mating-competent yeast cells can be acquired through alternative processes such as double strand breaks (DSBs) at the *MAT* loci and DNA replication errors. The common purpose, which is to generate hybrid strains, would be achieved by using either method.

In addition, CRISPR/Cas9 system [102,103] was used for artificial mating type switching through a double-strand break at the *MAT* locus. According to gRNA sequences, Cas9-mediated double strand breaks (DSBs) are generated at either Y**a** or Yα region [104]. Unlike mating type conversion using Ho endonuclease, it is possible to switch mating type of yeast strains lacking *HMR***a** and *HML*α by addition of the donor DNA segment. Similar to haploid cells, diploid cells were switched to the specified mating type (*MAT***a**/**a** or *MAT*α/α) using this approach [104]. As described above, mating-competent yeast cells can be acquired without going through sporulation (Figure 8). It might be a potential public concern that some of these methods require genetic modifications, unlike conventional crossbreeding. However, including genome editing with CRISPR/Cas9 system, all genetic modifications can be introduced into the yeast cells using episomal vectors, which allows exclusion of the exogenous DNA throughout the entire genome of crossbreeds after its removal.

## 6. Conclusions

Infertility challenges pose a significantly high challenge for yeast trait modification using conventional crossbreeding. There are numerous valuable yeast resources that cannot be fully exploited due to sporulation deficiency. In natural environments, deleterious mutations for sexual reproduction would have been purged from the yeast genome. Industrial yeasts used in modern human societies have acquired excellent characteristics in fermentation with partial sacrifice of sporulation efficiency, due to protection from harsh environmental changes. Although a variety of yeast strains have been generated during the long human history, the technique of conventional crossbreeding used for yeast trait modification based on the molecular mechanism has reached its limit. To meet the needs of future yeast strain development for each custom-engineered fermentation process, alternative techniques to bypass sporulation are essentially required to acquire mating-competent yeast cells. In this review, I have described several alternative methods to generate mating-competent cells from parental yeasts exhibiting severe deficiencies in sporulation. Even if there is a need to use exogenous genes, all genetic modifications described here can be introduced into the yeast cells using episomal vectors, which allows complete removal of the modifications following the isolation of crossbreds. These methods will enable us to improve yeast resources beyond the limitation of conventional crossbreeding by facilitating the generation of new yeast strains exhibiting desirable properties for industrial applications.

## Figures and Tables

**Table 1:** *S. cerevisiae* genes described in this review.

Gene	Description	Reference
*MAT*	mating type locus; **a** or α allele	[7,8]
*STE2*	seven transmembrane receptor for α-factor pheromone	[18]
*STE3*	seven transmembrane receptor for **a**-factor pheromone	[18]
*MFA1*	mating pheromone **a**-factor	[18]
*MFα1*	mating pheromone α-factor	[18]
*GPA1*	GTP-binding α subunit of heterotrimeric G-protein	[18,19]
*STE4*	β subunit of heterotrimeric G-protein	[18,19]
*STE18*	γ subunit of heterotrimeric G-protein	[18,19]
*IME1*	master regulator of meiosis	[20]
*RME1*	zinc finger protein involved in control of meiosis	[20]
*IRT1*	long noncoding RNA located in the *IME1* promoter	[21]
*HMR***a** *	silenced copy of **a** sequence	[22]
*HML*α	silenced copy of α sequence	[22]
*HO*	site-specific endonuclease for the *MAT* locus	[18]
*ASH1*	zinc-finger inhibitor of *HO* transcription	[23]
*SHE1*	type V myosin motor involved in actin-based transport	[24]
*SHE2*	RNA-binding protein that binds specific mRNAs	[24]
*SHE3*	protein adaptor between She1 and the She2-mRNA complex	[24]
*MSN2*	stress responsive transcriptional activator	[25,26]
*MSN4*	stress responsive transcriptional activator	[25,26]
*RIM15*	protein kinase involved in cell proliferation	[27]
*IME2*	serine/threonine protein kinase involved in activation of meiosis	[28,29]
*GIS1*	histone demethylase and transcription factor	[30]
*SPO11*	meiosis-specific protein that initiates meiotic recombination	[31]

* The “a”(shown in bold) means mating-type “a”.

**Table 2:** Mating type dependent gene expression.

Cell Type	Mating-Related Genes			Meiosis-Related Genes		
	*asg*	*αsg*	*hsg*	*RME1*	*IRT1*	*IME1*
**a** *	ON	OFF	ON	ON	ON	OFF
α	OFF	ON	ON	ON	ON	OFF
**a**/α	OFF	OFF	OFF	OFF	OFF	ON

* The “**a**”(shown in bold) means mating-type “**a**”.

## Abbreviations

***a**sg*	**a**-type-specific genes
*αsg*	α-type-specific genes
DSBs	double strand breaks
FACS	fluorescence-activated cell sorting
GPCR	G-protein coupled receptor
*hsg*	haploid-specific genes
LOH	loss of heterozygosity
MAPK	mitogen activated protein kinase
PDS	post-diauxic shift
PKA	protein kinase A
RNP	ribonucleoprotein particle
STRE	stress response element
TORC1	target of rapamycin complex I
URS	upstream repression sequence
